# Long-term mortality following complications after elective surgery: a secondary analysis of pooled data from two prospective cohort studies

**DOI:** 10.1016/j.bja.2022.06.019

**Published:** 2022-08-19

**Authors:** Alexander J. Fowler, Yize I. Wan, John R. Prowle, Michelle Chew, Douglas Campbell, Brian Cuthbertson, Duminda N. Wijeysundera, Rupert Pearse, Tom Abbott

**Affiliations:** 1 Faculty of Medicine and Dentistry, Queen Mary University of London, London, UK; 2 Department of Anaesthesia and Intensive Care, Biomedical and Clinical Sciences, Linköping University, Linköping, Sweden; 3 Department of Anaesthesia and Perioperative Medicine, Auckland City Hospital, Auckland, New Zealand; 4 Department of Anesthesiology and Pain Medicine, University of Toronto, Toronto, ON, Canada; 5 Department of Critical Care Medicine, Sunnybrook Health Sciences Centre, Toronto, ON, Canada; 6 Department of Anesthesia, St Michael's Hospital, Toronto, ON, Canada

**Keywords:** long-term survival, perioperative care, surgical complications, surgical outcomes, mortality

## Abstract

**Background:**

Complications after surgery affect survival and quality of life. We aimed to confirm the relationship between postoperative complications and death within 1 yr after surgery.

**Methods:**

We conducted a secondary analysis of pooled data from two prospective cohort studies of patients undergoing surgery in five high-income countries between 2012 and 2014. Exposure was any complication within 30 days after surgery. Primary outcome was death within 1 yr after surgery, ascertained by direct follow-up or linkage to national registers. We adjusted for clinically important covariates using a mixed-effect multivariable Cox proportional hazards regression model. We conducted a planned subgroup analysis by type of complication. Data are presented as mean with standard deviation (sd), *n* (%), and adjusted hazard ratios (aHRs) with 95% confidence intervals (CIs).

**Results:**

The pooled cohort included 10 132 patients. After excluding 399 (3.9%) patients with missing data or incomplete follow-up, 9733 patients were analysed. The mean age was 59 [sd 16.8] yr, and 5362 (55.1%) were female. Of 9733 patients, 1841 (18.9%) had complications within 30 days after surgery, and 319 (3.3%) died within 1 yr after surgery. Of 1841 patients with complications, 138 (7.5%) died within 1 yr after surgery compared with 181 (2.3%) of 7892 patients without complications (aHR 1.94 [95% CI: 1.53–2.46]). Respiratory failure was associated with the highest risk of death, resulting in six deaths amongst 28 patients (21.4%).

**Conclusions:**

Postoperative complications are associated with increased mortality at 1 yr. Further research is needed to identify patients at risk of complications and to reduce mortality.

More than 330 million surgical procedures are carried out worldwide every year.^1^ There is substantial variation in estimates of postoperative morbidity and mortality, in part because of heterogeneity in reporting of postoperative outcomes.^2^, ^3^, ^4^ In the UK, around five million surgical procedures are performed each year, after which 1% of patients die within 30 days.^5^^,^^6^ Most perioperative research studies use 30 day mortality as a marker of harm associated with surgery.^7^ However, there is growing evidence that mortality rates remain elevated between 30 and 90 days after surgery.^5^^,^^8^ Despite this, studies describing the patterns and determinants of long-term mortality after surgery are few in number.

Complications, such as infections, cardiovascular conditions, postoperative pulmonary complications, renal impairment, and so on, occur after one in every five surgical procedures.^9^, ^10^, ^11^, ^12^, ^13^ Evidence from a large multicentre cohort study in the USA identified strong association between postoperative medical complications and reduced long-term survival. However, whilst the study was groundbreaking at the time of publication, it only investigated eight surgical procedure categories and represents clinical practice from a single country conducted more than 20 yr ago.^14^ Thus, this study retains only limited relevance for contemporary surgical and perioperative practice, with restricted generalisability to the wider international surgical population.^1^^,^^13^ Previously, we examined the association between infection, the most common postoperative complication, and 30 day mortality.^10^^,^^15^ To plan delivery of care for patients undergoing surgery, we must better understand the long-term outcomes after complications, in addition to widely reported data describing short-term harms.

The primary aim of the study was to describe the crude and adjusted association between postoperative complications and death within 1 yr after surgery using data from two international, multicentre, observational cohort studies. The secondary aim was to measure the association between the severity of complications and 1 yr survival. Finally, we hypothesised that the rate of mortality would vary with type of complication.

## Methods

### Study design

This was a secondary analysis of data from two prospective observational cohort studies, which have been described previously. The International Surgical Outcomes Study (ISOS) was an international, multicentre cohort study of perioperative morbidity and mortality in patients undergoing elective inpatient surgery.^13^ Data collection occurred during a 7 day period in 2014. All adult patients admitted to participating centres for elective surgery were eligible. Patients undergoing day-case surgery or radiological procedures were excluded. This analysis was a subgroup of the core ISOS and included patients from Sweden, New Zealand, and England, where we collected mortality data up to 1 yr after surgery. In England, these data were collected with individual patient consent using civil registry data held by NHS Digital (REC: 18/YH/0310; Confidentiality Advisory Group: 18/CAG/0205; DSA:NIC-68740). In Sweden, death was identified from patient notes and confirmed with national registry data. In New Zealand, these data were collected from the Mortality Collection (Ministry of Health, New Zealand). The second cohort is derived from the Measurement of Exercise Tolerance before Surgery (METS) study, a prospective international observational cohort study of surgical outcomes in 25 hospitals across England, Canada, Australia, and New Zealand.^12^ Data collection occurred between 2012 and 2014. Eligible patients were aged ≥40 yr with at least one risk factor for vascular disease or cardiovascular complications and underwent elective inpatient noncardiac surgery. Follow-up data were collected for 1 yr after surgery from direct patient follow-up. The study was reviewed and approved by the South East Coast (Surrey) Research Ethics Committee (REC: 13/LO/0135). Both studies were conducted in accordance with the Research Governance Framework and the Declaration of Helsinki; a summary of each is in Supplementary Table S1. We developed a statistical analysis plan before starting this analysis and report findings in line with the Strengthening the Reporting of Observational Studies in Epidemiology (STROBE) and REporting of studies Conducted using Observational Routinely-collected Data (RECORD) guidance.^16^ No sample size calculation was performed for this secondary analysis of the two prospective studies.^17^

### Outcomes

The primary outcome measure was death at 1 yr after surgery. The secondary outcome was death within 30 days after surgery.

### Exposure

The exposure of interest was presence of any complication within 30 days after surgery. Complications were identified prospectively in both constituent studies. We grouped complications into infective (including surgical site infection, deep wound infection, and pneumonia), cardiovascular (including arrhythmia, myocardial infarction, and stroke), bleeding/thromboembolic (including pulmonary embolus and gastrointestinal bleeding), respiratory failure, acute kidney injury, reoperation, and others.^13^ The grouping of complications, stratified by study, is reported in Supplementary Table S2.

### Variables

Age was recorded in completed years at time of surgery. Sex was recorded as male or female. Smoking status was dichotomised as current smoker or not current smoker. Patients were classified according to the ASA physical status (PS) classification (ASA PS Grades 1, 2, 3, 4, and 5).^18^ We grouped surgical procedures into eight categories (vascular, thoracic, peritoneal, orthopaedics, obstetrics, urology/gynaecology, head & neck, and neurosurgery) based on the primary procedure performed. The use of a laparoscopic surgical technique, the severity of surgery, and cancer being the indication for surgery were each determined by investigators at time of data collection. We classified anaesthetic techniques as general anaesthesia alone, combined general anaesthesia and regional anaesthesia, regional anaesthesia alone, and sedation alone. We captured the following chronic diseases: cancer, coronary artery disease, diabetes mellitus, cardiac failure, stroke, and chronic obstructive pulmonary disease. We defined chronic kidney disease as an estimated glomerular filtration rate (eGFR) <60 ml min^−1^ (1.73 m)^−2^ using creatinine measured within 30 days before surgery. We calculated eGFR using the 2021 Chronic Kidney Disease Epidemiology Collaboration (CKD-EPI) creatinine equation, which does not require patient ethnicity to determine eGFR.^19^ Liver cirrhosis was captured only in ISOS, so we excluded this disease from statistical testing.

### Missing data

We excluded patients from the primary analysis for whom follow-up at 1 yr after surgery was incomplete (or linkage incomplete in England); we report their characteristics in Supplementary Table S3. We report the rate of missingness for all variables and determined the patterns. Data missing completely at random were handled in two ways: first, by complete case analysis for the primary analysis; second, by imputation of missing variables. Here, we used multiple imputation with chained equations for five imputed data sets and repeated our primary analysis.^20^^,^^21^

### Statistical methods

We dichotomised the patients according to presence or absence of complications within 30 days after surgery. We present the baseline characteristics, reported as number with percentage for categorical variables, and we report continuous data as mean (with standard deviation [sd]). We present the crude rate of death at both 30 and 365 days after surgery, stratified by the presence or absence of complications.

We present Kaplan–Meier survival plots for postoperative complications *vs* no complications, with the associated log-rank test statistic. Mixed-effects Cox proportional hazards modelling with country, nested within study, as a random intercept was used to account for the clustered nature of the data. We report univariable and multivariable (adjusted) analysis of complications and 1 yr survival, presented as hazard ratios (HRs) with 95% confidence intervals (CIs). Covariates were included based on prior knowledge of association with mortality and clinical significance. We included the following variables in the multivariable model: age, sex, ASA grade, smoking status, surgical procedure category, surgical severity, cancer surgery, all chronic diseases, and count of diseases.^8^^,^^11^^,^^12^^,^^22^, ^23^, ^24^, ^25^, ^26^, ^27^, ^28^ We explored the proportional hazards assumption using Schoenfeld residuals. We explored linearity of age by comparing a model, including age as untransformed variable, to a series of models with different transformations.

We identified a violation of the proportional hazards assumption for our primary exposure. This was handled *post hoc* using a time-step function and dividing the influence of complications into two time periods (0–20 and >20 days); we present the HRs for each time period. To determine the variability between nations and studies, we report the intra-class correlation coefficient (ICC). The ICC is the proportion of variability in outcome explained by the clustering structure. All analyses were performed using R (version 4.0.1). We used *data. table* for data manipulation, *survival* and *coxme* libraries for survival analysis, and the *mice* library for multiple imputation, and we generated figures using *ggplot 2*.

### Sensitivity analyses

In ISOS, complication severity was graded for each complication using a four-item scale, and in the METS study complications were recorded as either present or absent, with a grading for the most severe complication. We allocated each patient to a group based on the severity of their worst complication, and we measured the association between worst complication severity and subsequent 1 yr mortality. To describe the relationship between individual complications and 1 yr survival, we present the crude rate of 1 yr death and the HR for death associated with each complication type. For patients who suffered multiple complications, we included each patient in each analysis. To determine the duration of postoperative follow-up identifying most deaths, we report the cumulative rate of death over 1 yr after surgery, stratified by the presence of complications. We did a *post hoc* analysis exploring the relationship between anaesthetic technique, development of complications, and subsequent 1 yr mortality.

## Results

### Patient selection

**Fig 1:**
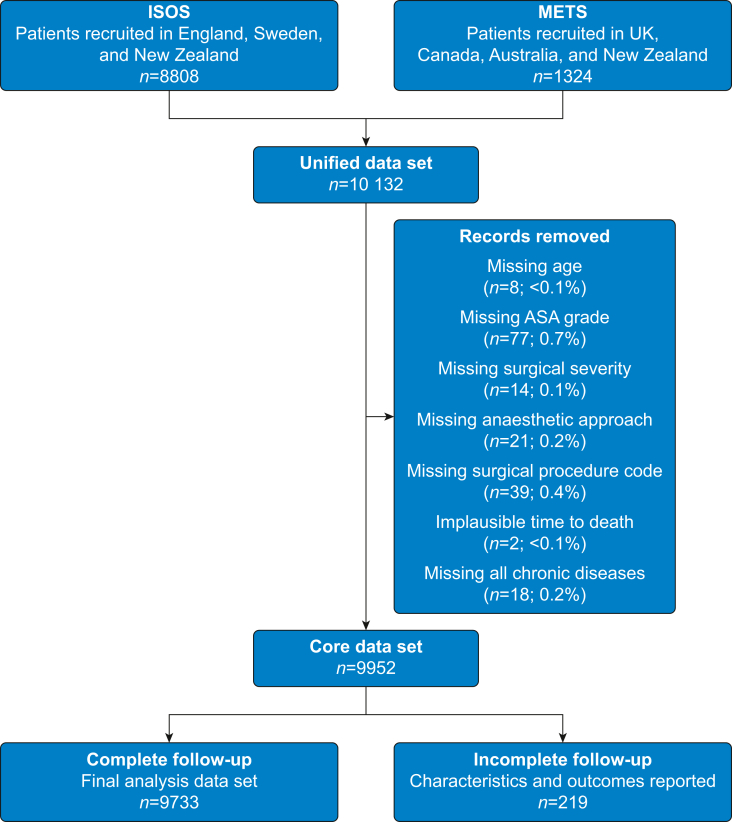
Flow diagram summarising patient inclusion. ISOS, International Surgical Outcomes Study; METS, Measurement of Exercise Tolerance before Surgery.

We included 10 132 patients, of whom 8808 were from ISOS and 1324 were from the METS study. After predefined exclusions, 9952 patients remained, of whom 9733 had complete follow-up data and were included in the analysis. The cohort selection process is outlined in Fig 1.

### Patient characteristics

The mean age was 59 [sd 16.8], and 5362 (55.1%) patients were female. Most patients were ASA PS Grade 2 (4991; 51.3%). The most common type of surgery was orthopaedics (2504; 25.5%), and 6609 (67.9%) procedures were performed under general anaesthesia (Table 1). The characteristics of patients with incomplete 1 yr follow-up are in Supplementary Table S3.

**Table 1:** Characteristics of patients, stratified by the presence of complications within 30 days of surgery. Data are presented as *n* (%) unless otherwise stated. ASA, American Society of Anesthesiologists; COPD, chronic obstructive pulmonary disease; IQR, inter-quartile range; sd, standard deviation.

Variable	All	Complications within 30 days	
		Present	Absent
*N*	9733	1841	7892
Age and sex			
Mean age, yr [sd]	59 [16.8]	64.7 [14.7]	57.7 [17]
Female sex	5362 (55.1)	846 (46)	4516 (57.2)
Male sex	4371 (44.9)	995 (54)	3376 (42.8)
ASA physical status			
1	2113 (21.7)	197 (10.7)	1916 (24.3)
2	4991 (51.3)	848 (46.1)	4143 (52.5)
3	2480 (25.5)	720 (39.1)	1760 (22.3)
4	149 (1.5)	76 (4.1)	73 (0.9)
Chronic diseases			
COPD	1464 (15)	318 (17.3)	1146 (14.5)
Diabetes mellitus	1254 (12.9)	306 (16.6)	948 (12)
Coronary artery disease	1200 (12.3)	370 (20.1)	830 (10.5)
Chronic kidney disease	1023 (10.5)	326 (17.7)	697 (8.8)
Cancer	953 (9.8)	329 (17.9)	624 (7.9)
Heart failure	271 (2.8)	99 (5.4)	172 (2.2)
Current smoker	1277 (13.1)	214 (11.6)	1063 (13.5)
Surgical procedure type			
Orthopaedics	2504 (25.7)	380 (20.6)	2124 (26.9)
Peritoneal	1698 (17.4)	494 (26.8)	1204 (15.3)
Obstetrics	1463 (15)	160 (8.7)	1303 (16.5)
Urological and gynaecological	1410 (14.5)	271 (14.7)	1139 (14.4)
Head and neck	904 (9.3)	111 (6)	793 (10)
Other	624 (6.4)	90 (4.9)	534 (6.8)
Breast	334 (3.4)	27 (1.5)	307 (3.9)
Thoracic	483 (5)	219 (11.9)	264 (3.3)
Vascular	308 (3.2)	87 (4.7)	221 (2.8)
Neurological	5 (0.1)	2 (0.1)	3 (0)
Surgical features			
Low severity	1113 (11.4)	80 (4.3)	1033 (13.1)
Moderate severity	4187 (43)	517 (28.1)	3670 (46.5)
High severity	4433 (45.5)	1244 (67.6)	3189 (40.4)
Surgery for cancer	2224 (22.9)	620 (33.7)	1604 (20.3)
Laparoscopic surgery	1761 (18.1)	326 (17.7)	1435 (18.2)
Anaesthetic technique			
General only	6609 (67.9)	1191 (64.7)	5418 (68.7)
Regional only	1869 (19.2)	254 (13.8)	1615 (20.5)
General with regional	1040 (10.7)	371 (20.2)	669 (8.5)
Sedation only	215 (2.2)	25 (1.4)	190 (2.4)
Study			
ISOS	8460 (86.9)	1503 (81.6)	6957 (88.2)
METS	1273 (13.1)	338 (18.4)	935 (11.8)
Nation			
UK	6929 (71.2)	1256 (68.2)	5673 (71.9)
New Zealand	1481 (15.2)	296 (16.1)	1185 (15)
Sweden	681 (7)	163 (8.9)	518 (6.6)
Canada	402 (4.1)	59 (3.2)	343 (4.3)
Australia	240 (2.5)	67 (3.6)	173 (2.2)

### Incidence of complications

Of 9733 patients, 1841 (18.9%) patients suffered complications within 30 days after surgery. Infections were the most common complication and affected 588 (6.0%) patients. The median age of patients who suffered complications was 67 yr (inter-quartile range [IQR]: 56–75) compared with 61 yr (IQR: 46–71) amongst patients who did not suffer complications. Some 995 (54.0%) of patients who suffered complications were male compared with 3376 (42.8%) of patients who did not. The prevalence of chronic diseases was greater amongst patients who suffered complications than those who did not (Table 1).

### Outcomes

Within 30 days after surgery, 35 of 10 132 patients (0.3%) died; within 1 yr after surgery, 319 patients died (3.3%). The characteristics of patients, stratified by survival at 1 yr after surgery, are in Supplementary Table S4.

### Association between complications and outcomes

**Fig 2:**
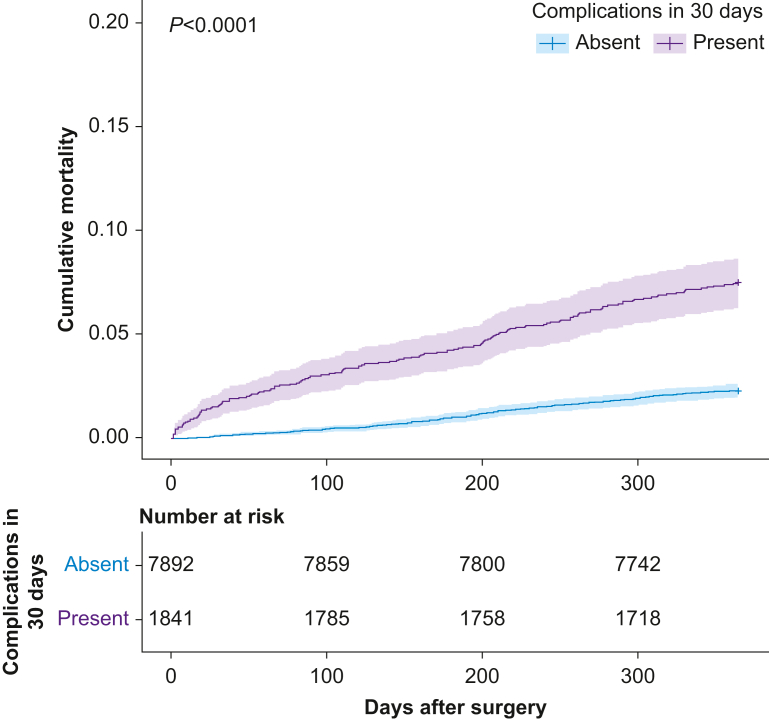
Cumulative mortality after surgery, stratified by the presence of complications within 30 days of surgery. *P*-value derived from a log-rank test.

**Fig 3:**
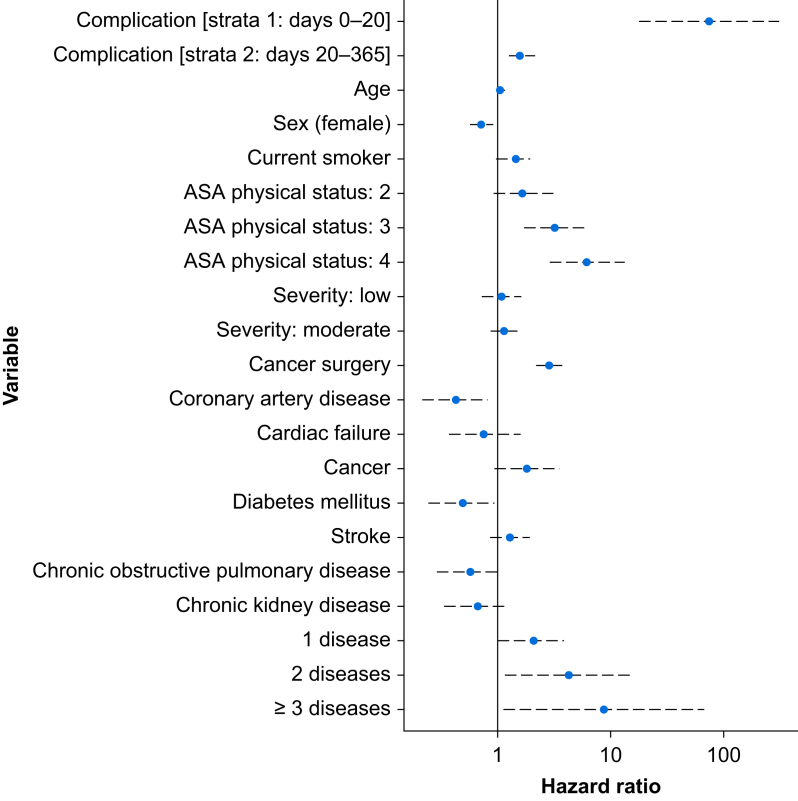
Hazard ratios from the multivariable, multilevel Cox proportional hazards regression model after stratifying complications to resolve non-proportionality. Dashed lines indicate associated 95% confidence intervals.

Amongst 1841 patients who suffered complications, 28 (1.5%) died within 30 days and 187 (7.5%) died within 1 yr after surgery. Amongst 7892 patients who did not suffer complications, seven (0.3%) died within 30 days and 181 (2.3%) died within 1 yr after surgery (Table 2; Fig 2). Before adjustment, the HR for death was 3.38 (95% CI: 2.71–4.22). After adjustment, the HR for death was 1.94 (95% CI: 1.53–2.46) (Fig 3). We explored including a non-linear term for age but found minimal improvement in log likelihood. We treated the presence of any complication as a time-varying variable with two steps (0–20 and >20 days) because of non-proportional hazards (Supplementary Fig. S1). These steps were selected based on review of Schoenfeld residual plots. After adjustment, the influence of complications was greatest in the first 20 days after surgery with HR 75.98 (17.93–322) that reduced to 1.61 (1.26–2.07) after 20 days. We observed variation between studies and nations; the ICC between nations was 0.08 and between studies was 0.265 (Supplementary Table S5). The greatest risk of death was amongst patients recruited to ISOS in England and lowest amongst those recruited to METS in Canada. The adjusted HRs associated with complications at different time points and for variables included in the multivariable models are reported in Table 3 and Supplementary Table S6. Our findings were unchanged when we used multiple imputation to derive a complete data set (Supplementary Table S7). We constructed a directed acyclic graph to examine causal paths between complications and subsequent 1 yr survival (Supplementary Fig. S2; code supplement A). The lack of data in the period between the end of the prospective studies and subsequent 1 yr mortality follow-up prevented any assessment of direct or indirect causal relationships.

**Table 2:** Rate of death and hazard ratio for death, presented by the presence/absence of complications within 30 days and specific complication groups. Patients may have suffered multiple complications, so the sum of specific complications will not equal the overall number of patients suffering a complication. Hazard ratios are presented with 95% confidence intervals (CIs) and were unadjusted.

	*N*	Deaths, *n* (%)		Unadjusted hazard ratio for death by 365 days (95% CI)
		30 days	365 days	
All patients	9733	35 (0.4)	319 (3.3)	—
Any complication				
Present	1841	28 (1.5)	138 (7.5)	3.38 (2.71–4.22)
Absent	7892	7 (0.1)	181 (2.3)	Reference
Specific complication groups				
Infection	588	8 (1.4)	65 (11.1)	4.19 (3.19–5.5)
Cardiac	286	18 (6.3)	38 (13.3)	4.86 (3.46–6.81)
Acute kidney injury	247	6 (2.4)	20 (8.1)	2.66 (1.69–4.19)
Bleed or thromboembolism	308	5 (1.6)	28 (9.1)	3.05 (2.07–4.49)
Respiratory failure	28	2 (7.1)	6 (21.4)	7.96 (3.55–17.86)
Surgical requiring reoperation	348	8 (2.3)	36 (10.3)	3.60 (2.55–5.1)
Other complications	540	4 (0.7)	26 (4.8)	1.53 (1.02–2.28)
Severity of worst complication				
Mild	736	1 (0.1)	27 (3.7)	1.6 (1.1–2.4)
Moderate	864	1 (0.1)	60 (6.9)	3.1 (2.3–4.1)
Severe	194	1 (0.5)	25 (12.9)	6.0 (4.0–9.1)
Death	25	25 (100)	25 (100)	—

**Table 3:** Variables included in the multivariable adjusted, multilevel Cox proportional hazards model before time-varying stratification of complication. Random effects components were country nested within study, and the value represents the standard deviation of the intercept for the log hazard ratio. ∗*P*-value <0.05. *P*-value for global Schoenfeld test=0.03. ASA, American Society of Anesthesiologists; CI, confidence interval; COPD, chronic obstructive pulmonary disease.

Feature	Adjusted hazard ratio (95% CI)	*z*
Complication within 30 days	1.94 (1.53–2.46)	5.55∗
Age (1 yr increment)	1.04 (1.03–1.05)	7.38∗
Sex: female *vs* male	0.73 (0.58–0.93)	–2.98∗
ASA physical status: 2 *vs* 1	1.63 (0.9–2.95)	1.64
ASA physical status: 3 *vs* 1	3.15 (1.71–5.8)	3.72∗
ASA physical status: 4 *vs* 1	6.11 (2.85–13.13)	4.72∗
Smoking status: current *vs* not current	1.45 (1.04–2.02)	6.2∗
Operative severity: low *vs* high	1.07 (0.73–1.56)	0.41
Operative severity: moderate *vs* high	1.15 (0.89–1.48)	1.07
Surgery for cancer	2.89 (2.24–3.72)	8.17∗
Coronary artery disease	0.36 (0.17–0.74)	–2.58∗
Cardiac failure	0.65 (0.3–1.38)	–0.84
Cancer	1.59 (0.78–3.23)	1.72
Diabetes mellitus	0.42 (0.2–0.86)	–2.14∗
Stroke	1.25 (0.84–1.86)	1.08
COPD	0.48 (0.23–1)	–1.67
Chronic kidney disease	0.59 (0.29–1.23)	–1.23
Number of diseases: 1 *vs* 0	2.53 (1.21–5.28)	2.10∗
Number of diseases: 2 *vs* 0	5.64 (1.4–22.68)	2.24∗
Number of diseases: ≥3 *vs* 0	15.33 (1.72–136.85)	2.15∗
**Random effects components** Study/country Country	**Standard deviation** 0.65 1.20	**Variance** 0.42 1.45

### Sensitivity analyses

To explore the relationship between the severity of complications and 1 yr survival, we did a sensitivity analysis amongst the subgroup of patients who had suffered any complication, stratifying patients by the severity of their worst complication. Amongst 1841 patients who suffered complications, the worst complication was graded minor amongst 736 (40.0%), moderate amongst 774 (46.9%), and severe amongst 194 (10.5%). Twenty-five patients died during their hospital admission after complications. There was a clear graduation of effect in long-term outcomes according to severity of complication, with some 25 (12.9%) patients who suffered severe complications dying within 1 yr (HR 6.0 [95% CI: 4.0–9.1]) (Table 2).

The complication associated with the greatest crude rate of death within 1 yr after surgery was respiratory failure, resulting in six deaths amongst 28 patients (21.4%; 95% CI: 8.3–41.0%) (Supplementary Fig. S2). The complication associated with the lowest crude rate of death within 1 yr after surgery was ‘other’ complications (26 of 540; 4.8%). Given the low number of events when stratified by type of complication, we did not perform multivariable adjustment in this sensitivity analysis (Table 2; Supplementary Fig. S3).

To identify optimal duration of follow-up amongst patients after surgery, we did a *post hoc* analysis to demonstrate when deaths occur, stratified by the presence or absence of complications (Supplementary Fig. S4). Amongst patients with complications, one quarter of deaths occurring in the year after surgery occurred within the first 38 days after surgery, compared with the first 145 days after surgery for patients who did not suffer complications (Supplementary Table S8).

We did a *post hoc* exploratory analysis of the association between different anaesthesia techniques and subsequent risk of complications and death (Supplementary Table S9). Mixed-modality anaesthesia was associated with the greatest risk of complications (odds ratio: 2.52 [95% CI: 2.19–2.91]). There was no significant association between anaesthesia technique and survival after including it in the final multivariable, multilevel Cox proportional hazards model. In addition, there was no improvement in model as measured using analysis of variance (*P*=0.31).

## Discussion

The principal finding of this analysis of two observational cohort studies across five nations is that complications in the 30 days after elective surgery are associated with a two-fold increase in 1 yr mortality. Infections were the most common complications affecting one in 20 patients, of whom 11% died within the year after surgery. The complication associated with the greatest risk of 1 yr death was respiratory failure, where one in five patients died. Our findings persisted after adjustment for important patient-level characteristics and for the different study/country combinations included in the data set.

Our finding that complications are associated with reduced long-term survival is consistent with data from a multicentre cohort study in the USA, first published in 2005.^14^ The authors reported a substantial reduction in survival up to 8 yr after surgery amongst patients who sustained complications. However, the study cohort inclusion window ended in 1999, and the authors reported an overall 1 yr mortality rate of 6.9% amongst patients without complications, which is much greater than would be expected of contemporary surgical practice, reflected by a three-fold lower mortality rate in our cohort. In contrast to Khuri and colleagues,^14^ we observed a 60% increase in the relative hazard of death over the time periods of the two studies, which suggests that whilst the overall risk of death has successfully been reduced overall, the relative influence of complications remains profound. We also identified a strong time-dependent association between complications and survival, with a very high rate of death in the first 20 days after surgery, followed by a lower but still elevated risk of death amongst patients who suffered complications, which persisted after adjusting for confounding factors.

Death after suffering a complication has been described as failure to rescue. The incidence of failure to rescue is subject to substantial inter-hospital variation.^29^ We identified a similar variation in 1 yr survival amongst patients who suffered complications. Our modelling suggests that 8% of the variability in the rate of 1 yr death after surgery is explained by country. Whilst these findings may be sensitive to differing inclusion criteria or outcome definition between studies, they are likely to represent true variability that has several possible explanations. There may be between-country variation in selection criteria for surgery, prevalence of risk factors, and end-of-life practices.^30^ Upcoming studies, such as the Latin American Surgical Outcomes Study, will help understand these potential variations.^31^ Differences in the management of complications may also alter the likelihood of long-term survival. For example, there is growing evidence of association between repeated acute cellular injury and chronic harm in several organ systems, including kidney injury, myocardial injury, and cognitive dysfunction.^9^^,^^26^^,^^27^^,^^32^ However, the optimal way to improve long-term survival for patients who have suffered complications remains unclear. Future research could focus on individual types of complications or organ systems, for example the influence of interventions reducing the progression of chronic kidney disease and cardiac risk factors amongst survivors of acute kidney injury.^33^

We hypothesised that risk of mortality may vary by type of complication. Respiratory failure, whilst rare, was associated with the greatest risk of postoperative mortality. However, clinical strategies to prevent postoperative pulmonary complications remain elusive. Recent studies have failed to find benefit with routine postoperative CPAP or noninvasive ventilation to prevent pneumonia and respiratory failure.^34^^,^^35^ Infective complications were the most common, affecting one in 20 patients, and were associated with a one in 10 rate of death after 1 yr. However, growing evidence suggests only marginal benefit from liberal antimicrobial prophylaxis to prevent postoperative infection.^36^ Further research is needed to inform decision-making around routine preventative antimicrobial therapy and to develop alternative strategies to reduce total antimicrobial use. During the COVID-19 pandemic, reports have highlighted the very poor short-term outcomes experienced by patients with perioperative SARS-CoV-2 infection.^8^^,^^32^ Our findings suggest that there is likely to be a persistently increased risk of death in this patient group. We observed that patients with severe complications in the immediate postoperative period had higher rates of death in the year after surgery. Both the type and severity of complications are therefore important in determining the impact on long-term survival. Amongst survivors of complications, there are broader negative effects, such as reduced mobility and loss of function, not captured by our data. Whilst we found that the rate of complications was significantly influenced by anaesthesia technique, there was no association after adjustment for other factors. This is likely because major abdominal or thoracic procedures may require mixed anaesthetic, which therefore acts as a confounder. One quarter of deaths in the year after surgery occur within 38 days amongst patients who suffer complications compared with 145 days amongst those who do not suffer complications. This suggests that studies reporting short-term measures of mortality are likely to underestimate the influence of complications. This problem may be exacerbated by the legal definition of a surgical complication in different countries, which may lead to a failure to recognise that a life-limiting complication has occurred.

Our analysis has several strengths. First, we included a large cohort of patients undergoing a wide range of surgical procedures across five nations, which makes our results widely generalisable to perioperative practice in high-income countries. Second, we used linkage to national death registry data to robustly determine postoperative death, with a low rate of missing data (<2%). Third, we incorporated data from two large, prospective cohort studies. Both had detailed and standardised data collection approaches with comparable definitions. Fourth, we did a detailed survival analysis, which was pre-specified and included handling of non-proportional hazards.

The analysis also has limitations. First, despite similarities between the studies, there were some variables, such as liver cirrhosis, which were present in only one study. In addition, there were small differences in the inclusion criteria of each study. We accounted for this and potential differences in patients within studies by using a mixed-effects model. By including a broader range of patients across multiple nations, the external generalisability of our study is improved, and between-study differences are unlikely to be differential by complication status and therefore would not bias our results. Second, a small number of patients did not have 1 yr outcome data. These patients were typically younger, with a lower burden of chronic disease (high proportion of ASA PS Grade 1) and a low incidence of postoperative complications. It is possible that some of these patients emigrated, in which case any subsequent death would not be captured in national registry data. However, this is unlikely to vary between patients with or without complications, and therefore unlikely to impact our findings. Third, any observational cohort study is prone to unmeasured confounding. We sought to control for sources of confounding through multivariable modelling. However, there may be variables not captured during the study that may influence our findings, such as events occurring between the end of the cohort-study data collection window and 1 yr following, and variable end-of-life practices.^30^ We did not use routinely collected data to capture diagnostic codes; this may have resulted in greater variability in clinical judgement and decreased the prevalence of certain conditions, such as cardiovascular diseases. Fourth, there are likely to be a wide variety of negative effects associated with complications, such as increased frailty and loss of function, that we did not capture but that are important to patients.

Patients with postoperative complications have a 60% increased risk of death during the first year after surgery compared with patients without complications. Respiratory failure, even before the COVID-19 pandemic, was most strongly associated with 1 yr mortality. Future studies should collect data over longer time periods to understand the relationship between short-term harm (complications), late effects, and subsequent patient outcomes. Whilst 1 yr survival is a useful measure of poor outcome, wider implications, such as quality of life and functional capacity, should also be explored. Further research is required to find out if it is possible to reduce the incidence of postoperative complications and to determine strategies to effectively treat complications to prevent poor long-term outcomes.

## Authors' contributions

Study design: TA, AJF, RP.

Data collection: AJF, TA, JRP, RP, MC, DC, DNW, BC.

Data analysis: AJF, TA.

Data interpretation: AJF, TA.

Writing of first draft of the article: AJF, TA.

Revising of the article for important intellectual content: all authors.

Approval of final version of the article: all authors.

AJF and TA had full access to the data and act as guarantors.
