# Supplementary material for: Long-term mortality following complications after elective surgery: a secondary analysis of pooled data from two prospective cohort studies
Source: Br J Anaesth. 2022 Aug 19;129(4):588–97. doi: 10.1016/j.bja.2022.06.019 (PMC9575043; doi:10.1016/j.bja.2022.06.019)
Supplement: Multimedia component 2 [file mmc2.docx]

**Long-term survival after surgery: a secondary analysis of pooled data from prospective cohort studies.**

**SUPPLEMENTARY DATA FILE**

| **Feature** | **ISOS** | **METS** |
| --- | --- | --- |
| Number of centers | 474 | 25 |
| Countries | 27 (Australia, Austria, Belgium, Brazil, Canada, China, Denmark, France, Germany, Greece, Hong Kong, Indonesia, Italy, Malaysia, The Netherlands, New Zealand, Nigeria, Portugal, Romania, Russia, South Africa, Spain, Sweden, Switzerland, Uganda, UK, and USA) | 4 (Australia, Canada, New Zealand, United Kingdom) |
| Inclusion criteria | Adult patients (age ≥18 years) undergoing surgery with overnight stay in hospital. | Age ≥ 40 years undergoing elective non-cardiac surgery under general/regional anaesthesia with overnight stay in hospital, with one risk factor for cardiac complications or coronary artery disease.* |
| Exclusion criteria | Emergency surgery, day-case surgery, radiological procedures. | No risk factors for cardiac complications or coronary artery disease. |
| Year(s) of data collection | April – August 2014 | 1 March 2013 – 25 March 2016 |
| Primary outcome | In-hospital complications within 30 days | Death or myocardial infarction within 30 days |
| Length of follow up | 30 days main cohort; 1 year in selected nations for this analysis. | 30 days and 1 year |

**Supplementary table 1. Summary of study characteristics.** Specific criteria listed in supplementary appendix to the original METS paper (Wijeysundera et al. Assessment of functional capacity before major non-cardiac surgery: an international, prospective cohort study. *Lancet* 2018;391(10140):2631-2640) - [1-s2.0-S0140673618311310-mmc1.pdf (els-cdn.com)](https://ars.els-cdn.com/content/image/1-s2.0-S0140673618311310-mmc1.pdf)

| **Complication group** | **ISOS** | **METS** |
| --- | --- | --- |
| Infection | Superficial surgical site infection, deep surgical site infection, body cavity infection, pneumonia, urinary tract infection, bloodstream infection | Surgical site infection, pneumonia |
| Cardiac | Myocardial infarction, arrhythmia, pulmonary oedema, stroke, cardiac arrest | Transient ischaemic accident, myocardial infarction, non-fatal cardiac arrest, heart failure |
| Bleeding/VTE | Bleeding, gastro-intestinal bleeding, pulmonary embolism | Bleeding, Deep vein thrombosis, pulmonary embolism |
| Respiratory failure | Acute respiratory distress syndrome | Acute respiratory distress syndrome |
| Acute Kidney injury | Acute kidney injury | Acute kidney injury* |
| Reoperation | Surgical or radiological procedure | Surgical procedure |

**Supplementary table 2. Complications from each study contributing to each complication group. ^*^**Acute kidney injury in METS was determined using KDIGO-AKI criteria using creatinine measurements made within the first three days after surgery.

| **Variable** | **Incomplete follow up** | **Complete follow up** |
| --- | --- | --- |
| N | 219 | 9733 |
| *Age & Sex* | | |
| Mean age (SD) | 57.3 (16.2) | 59 (16.8) |
| Female sex | 102 (46.6%) | 5362 (55.1%) |
| Male sex | 117 (53.4%) | 4371 (44.9%) |
| *ASA-PS grade* | | |
| I | 52 (23.7%) | 2113 (21.7%) |
| II | 129 (58.9%) | 4991 (51.3%) |
| III | 37 (16.9%) | 2480 (25.5%) |
| IV | 1 (0.5%) | 149 (1.5%) |
| *Chronic diseases* | | |
| COPD | 31 (14.2%) | 1464 (15%) |
| Diabetes mellitus | 30 (13.7%) | 1254 (12.9%) |
| Coronary artery disease | 24 (11%) | 1200 (12.3%) |
| Chronic kidney disease | 16 (7.3%) | 1023 (10.5%) |
| Cancer | 3 (1.4%) | 953 (9.8%) |
| Heart Failure | 5 (2.3%) | 271 (2.8%) |
| Current smoker | 31 (14.2%) | 1277 (13.1%) |
| *Surgical procedure type* | | |
| Orthopaedics | 70 (32%) | 2504 (25.7%) |
| Peritoneal | 32 (14.6%) | 1698 (17.4%) |
| Obstetrics | 34 (15.5%) | 1463 (15%) |
| Urology & Gynaecological | 35 (16%) | 1410 (14.5%) |
| Head & Neck | 13 (5.9%) | 904 (9.3%) |
| Other | 17 (7.8%) | 624 (6.4%) |
| Breast | 7 (3.2%) | 334 (3.4%) |
| Thoracic | 7 (3.2%) | 483 (5%) |
| Vascular | 4 (1.8%) | 308 (3.2%) |
| *Surgical features* | | |
| Low severity | 21 (9.6%) | 1113 (11.4%) |
| Moderate severity | 109 (49.8%) | 4187 (43%) |
| High severity | 89 (40.6%) | 4433 (45.5%) |
| Surgery for cancer | 38 (17.4%) | 2224 (22.9%) |
| Laparoscopic surgery | 43 (19.6%) | 1761 (18.1%) |
| *Anaesthetic technique* | | |
| General only | 150 (68.5%) | 6609 (67.9%) |
| Regional only | 44 (20.1%) | 1869 (19.2%) |
| General with regional | 18 (8.2%) | 1040 (10.7%) |
| Sedation only | 7 (3.2%) | 215 (2.2%) |
| *Study* | | |
| ISOS | 218 (99.5%) | 8460 (86.9%) |
| METS | 1 (0.5%) | 1273 (13.1%) |
| *Nation* | |  |
| New Zealand | 2 (0.9%) | 1481 (15.2%) |
| Sweden | 1 (0.5%) | 681 (7%) |
| UK | 216 (98.6%) | 6929 (71.2%) |

**Supplementary table 3. Characteristics of patients with incomplete follow up, stratified by the presence of complications within 30 days of surgery.** Data are presented as n (%) unless otherwise stated. SD; standard deviation, ASA-PS; American Society of Anesthesiologists Physical Status classification grading, COPD; Chronic obstructive pulmonary disease.

| **Variable** | **All** | **One year status** | | |
| --- | --- | --- | --- | --- |
|  |  | Non-Survivors | | Survivors |
| N | 9733 | 319 | | 9414 |
| *Age & Sex* | | | | |
| Mean age (SD) | 59 (16.8) | 71.6 (12.6) | 58.6 (16.8) | |
| Female sex | 5362 (55.1%) | 120 (37.6%) | 5242 (55.7%) | |
| Male sex | 4371 (44.9%) | 199 (62.4%) | 4172 (44.3%) | |
| *ASA-PS grade* | | | | |
| I | 2113 (21.7%) | 13 (4.1%) | 2100 (22.3%) | |
| II | 4991 (51.3%) | 120 (37.6%) | 4871 (51.7%) | |
| III | 2480 (25.5%) | 167 (52.4%) | 2313 (24.6%) | |
| IV | 149 (1.5%) | 19 (6%) | 130 (1.4%) | |
| *Chronic diseases* | | | | |
| COPD | 1464 (15%) | 67 (21%) | 1397 (14.8%) | |
| Diabetes mellitus | 1254 (12.9%) | 58 (18.2%) | 1196 (12.7%) | |
| Coronary artery disease | 1200 (12.3%) | 71 (22.3%) | 1129 (12%) | |
| Chronic kidney disease | 1023 (10.5%) | 88 (27.6%) | 1173 (12.5%) | |
| Cancer | 953 (9.8%) | 97 (30.4%) | 856 (9.1%) | |
| Heart Failure | 271 (2.8%) | 27 (8.5%) | 244 (2.6%) | |
| Current smoker | 1277 (13.1%) | 44 (13.8%) | 1233 (13.1%) | |
| *Surgical procedure type* | | | | |
| Orthopaedics | 2504 (25.7%) | 34 (10.7%) | 2470 (26.2%) | |
| Peritoneal | 1698 (17.4%) | 80 (25.1%) | 1618 (17.2%) | |
| Obstetrics | 1463 (15%) | 19 (6%) | 1444 (15.3%) | |
| Urology & Gynaecological | 1410 (14.5%) | 65 (20.4%) | 1345 (14.3%) | |
| Head & Neck | 904 (9.3%) | 40 (12.5%) | 864 (9.2%) | |
| Other | 624 (6.4%) | 24 (7.5%) | 600 (6.4%) | |
| Breast | 334 (3.4%) | 4 (1.3%) | 330 (3.5%) | |
| Thoracic | 483 (5%) | 31 (9.7%) | 452 (4.8%) | |
| Vascular | 308 (3.2%) | 22 (6.9%) | 286 (3%) | |
| Neurological | 5 (0.1%) | 0 (0%) | 5 (0.1%) | |
| *Surgical features* | | | | |
| Low severity | 1113 (11.4%) | 36 (11.3%) | 1077 (11.4%) | |
| Moderate severity | 4187 (43%) | 109 (34.2%) | 4078 (43.3%) | |
| High severity | 4433 (45.5%) | 174 (54.5%) | 4259 (45.2%) | |
| Surgery for cancer | 2224 (22.9%) | 182 (57.1%) | 2042 (21.7%) | |
| Laparoscopic surgery | 1761 (18.1%) | 49 (15.4%) | 1712 (18.2%) | |
| *Anaesthetic technique* | | | | |
| General only | 6609 (67.9%) | 207 (64.9%) | 6402 (68%) | |
| Regional only | 1869 (19.2%) | 41 (12.9%) | 1828 (19.4%) | |
| General with regional | 1040 (10.7%) | 57 (17.9%) | 983 (10.4%) | |
| Sedation only | 215 (2.2%) | 14 (4.4%) | 201 (2.1%) | |
| *Study* | | | | |
| ISOS | 8460 (86.9%) | 291 (91.2%) | 8169 (86.8%) | |
| METS | 1273 (13.1%) | 28 (8.8%) | 1245 (13.2%) | |
| *Nation* | | | | |
| United Kingdom | 6929 (71.2%) | 256 (80.3%) | 6673 (70.9%) | |
| New Zealand | 1481 (15.2%) | 20 (6.3%) | 1461 (15.5%) | |
| Sweden | 681 (7%) | 33 (10.3%) | 648 (6.9%) | |
| Canada | 402 (4.1%) | 2 (0.6%) | 400 (4.2%) | |
| Australia | 240 (2.5%) | 8 (2.5%) | 232 (2.5%) | |

**Supplementary table 4. Characteristics of patients, stratified by one year survivorship status.** Data are presented as n (%) unless otherwise stated. SD; standard deviation, IQR; inter-quartile range, ASA-PS; American Society of Anesthesiologists Physical Status classification grading, COPD; Chronic obstructive pulmonary disease.

| **Study : Nation** | **Relative risk of death at one year** |
| --- | --- |
| ISOS : New Zealand | 0.56 |
| ISOS : Sweden | 1.31 |
| ISOS : United Kingdom | 1.59 |
| METS : Australia | 1.35 |
| METS : Canada | 0.51 |
| METS : New Zealand | 0.81 |
| METS : UK | 1.44 |

**Supplementary table 5. Relative risk of death between nation/study combinations**. Derived from the exponent of the random effect components in the final multi-variable Cox proportional hazards model.

| **Feature** | **Adjusted hazard ratio (95% CI)** | **z** |
| --- | --- | --- |
| Complication within 30 days: |  |  |
| Hazard in days 0 – 20 | 75.98 (17.93 to 322) | 5.89* |
| Hazard in days 20 – 365 | 1.61 (1.26 to 2.07) | 3.8* |
| Age (one year increment) | 1.04 (1.03 - 1.05) | 7.35* |
| Sex: female vs. Male | 0.70 (0.56 - 0.89) | -2.95* |
| ASA-PS grade: II vs. I | 1.65 (0.91 to 2.98) | 1.65 |
| ASA-PS grade: III vs. I | 3.19 (1.73 to 5.82) | 3.72* |
| ASA-PS grade: IV vs. I | 6.02 (2.8 to 12.94) | 4.67* |
| Smoking status: current vs. not current | 1.45 (1.04 to 2.01) | 2.17* |
| Operative severity: low vs. high. | 1.06 (0.72 to 1.55) | 0.37 |
| Operative severity: moderate vs. high. | 1.14 (0.88 to 1.47) | 1.01 |
| Surgery for cancer | 2.88 (2.23 to 3.71) | 8.15* |
| Coronary artery disease | 0.42 (0.21 to 0.82) | -2.51* |
| Cardiac failure | 0.75 (0.36 to 1.53) | -0.78 |
| Cancer | 1.81 (0.93 to 3.54) | 1.75 |
| Diabetes mellitus | 0.48 (0.25 to 0.96) | -2.09* |
| Stroke | 1.24 (0.84 to 1.85) | 1.07 |
| COPD | 0.56 (0.28 to 1.12) | -1.65 |
| Chronic kidney disease | 0.66 (0.33 to 1.31) | -1.18 |
| Number of diseases: 1 vs. 0 | 2.08 (1.0. to 4.18) | 2.06* |
| Number of diseases: 2 vs. 0 | 4.31 (1.17 to 15.91) | 2.20* |
| Number of diseases: ≥3 vs. 0 | 8.91 (1.14 to 69.3) | 2.09* |
|  |  |  |
| **Random effects components:**  Study / Country  Country | **Standard deviation**  0.65  1.20 | **Variance**  0.42  1.45 |

**Supplementary table 6. Variables included in the multivariable adjusted, multi-level Cox proportional hazards model after stratifying complications to resolve non-proportionality.** Random effects components were country nested within study and the value represents the standard deviation of the intercept for the log hazard ratio. * p value < 0.05. P value for global Schoenfeld test = 0.03.

| **Feature** | **Adjusted hazard ratio (95% CI)** | **z** |
| --- | --- | --- |
| Complication within 30 days (stratified) |  |  |
| Days 0 – 20 | 82.97 (19.66 to 350.15) | 6.01* |
| Days 20 – 365 | 1.58 (1.24 to 2.03) | 3.65* |
| Age (one year increment) | 1.04 (1.03 to 1.05) | 7.73* |
| Sex: female vs. Male | 0.69 (0.55 to 0.87) | -3.18* |
| ASA-PS grade: II vs. I | 1.51 (0.85 to 2.69) | 1.41 |
| ASA-PS grade: III vs. I | 3.05 (1.69 to 5.51) | 3.70* |
| ASA-PS grade: IV vs. I | 5.98 (2.85 to 12.57) | 4.72* |
| Smoking status: current vs. not current | 1.44 (1.04 to 2) | 2.20* |
| Operative severity: low vs. high. | 1.07 (0.74 to 1.56) | 0.37 |
| Operative severity: moderate vs. high. | 1.17 (0.91 to 1.49) | 1.23 |
| Surgery for cancer | 2.77 (2.16 to 3.55) | 8.01* |
| Coronary artery disease | 0.43 (0.22 to 0.85) | -2.43* |
| Cardiac failure | 0.72 (0.35 to 1.47) | -0.90 |
| Cancer | 1.79 (0.92 to 3.5) | 1.71 |
| Diabetes mellitus | 0.49 (0.25 to 0.96) | -2.09 |
| Stroke | 1.18 (0.79 to 1.76) | 0.82 |
| COPD | 0.56 (0.28 to 1.11) | -1.67 |
| Chronic kidney disease | 0.68 (0.35 to 1.35) | -1.09 |
| Number of diseases: 1 vs. 0 | 2.09 (1.04 to 4.2) | 2.08* |
| Number of diseases: 2 vs. 0 | 4.43 (1.21 to 16.29) | 2.24* |
| Number of diseases: ≥3 vs. 0 | 8.48 (1.09 to 65.92) | 2.04* |
|  |  |  |
| **Random effects components:**  Study / Country  Country | **Standard deviation**  0.59  1.13 | **Variance**  0.36  1.29 |

**Supplementary table 7. Variables included in the multivariable adjusted, multi-level Cox proportional hazards model after stratifying complications to resolve non-proportionality using a complete dataset generated using multiple imputation.** Random effects components were country nested within study and the value represents the standard deviation of the intercept for the log hazard ratio. * p value < 0.05. P value for global Schoenfeld test = 0.03.

| **Proportion of all deaths in one year** | **All** | **Complications** | |
| --- | --- | --- | --- |
|  |  | **Absent** | **Present** |
| 25% | 85 days | 131 days | 38 days |
| 50% | 182 days | 197 days | 145 days |
| 75% | 260 days | 269 days | 244 days |

**Supplementary table 8. Time by which certain proportions of all deaths within one year of surgery were achieved, stratified by the presence or absence of complications within 30 days of surgery.**

|  |  | **Complications** | | **One year death**  **Number dead in one year of N (%)** | | **Adjusted hazard ratio^+^ (95% CI)** |
| --- | --- | --- | --- | --- | --- | --- |
| **Anaesthetic approach** | **N** | **N (%)** | **Odds Ratio* (95% CI)** | **Complications** | **No complications** |  |
| General | 6609 | 1191 (18.0) | Ref | 78 of 1191  (6.5) | 129 of 5418  (2.4) | Ref |
| Regional | 1869 | 1869 (13.6) | 0.72  (0.62 – 0.83) | 15 of 254  (5.9) | 26 of 1615  (1.6) | 0.91  (0.64 – 1.29) |
| General & Regional | 1040 | 371 (35.7) | 2.52  (2.19 – 2.91) | 41 of 371  (11.1) | 16 of 669  (2.4) | 1.16  (0.84 – 1.59) |
| Sedation | 215 | 25 (11.6) | 0.60  (0.39 – 0.91) | 4 of 25  (16.0) | 10 of 190  (5.3) | 1.57  (0.88 – 2.79) |

**Supplementary table 9. Anaesthesia type and rate of complications.** * Odds ratio of suffering a complication. + Hazard ratio for death within one year drawn from a re-run of the final multivariable, multi-level Cox proportional hazards model.


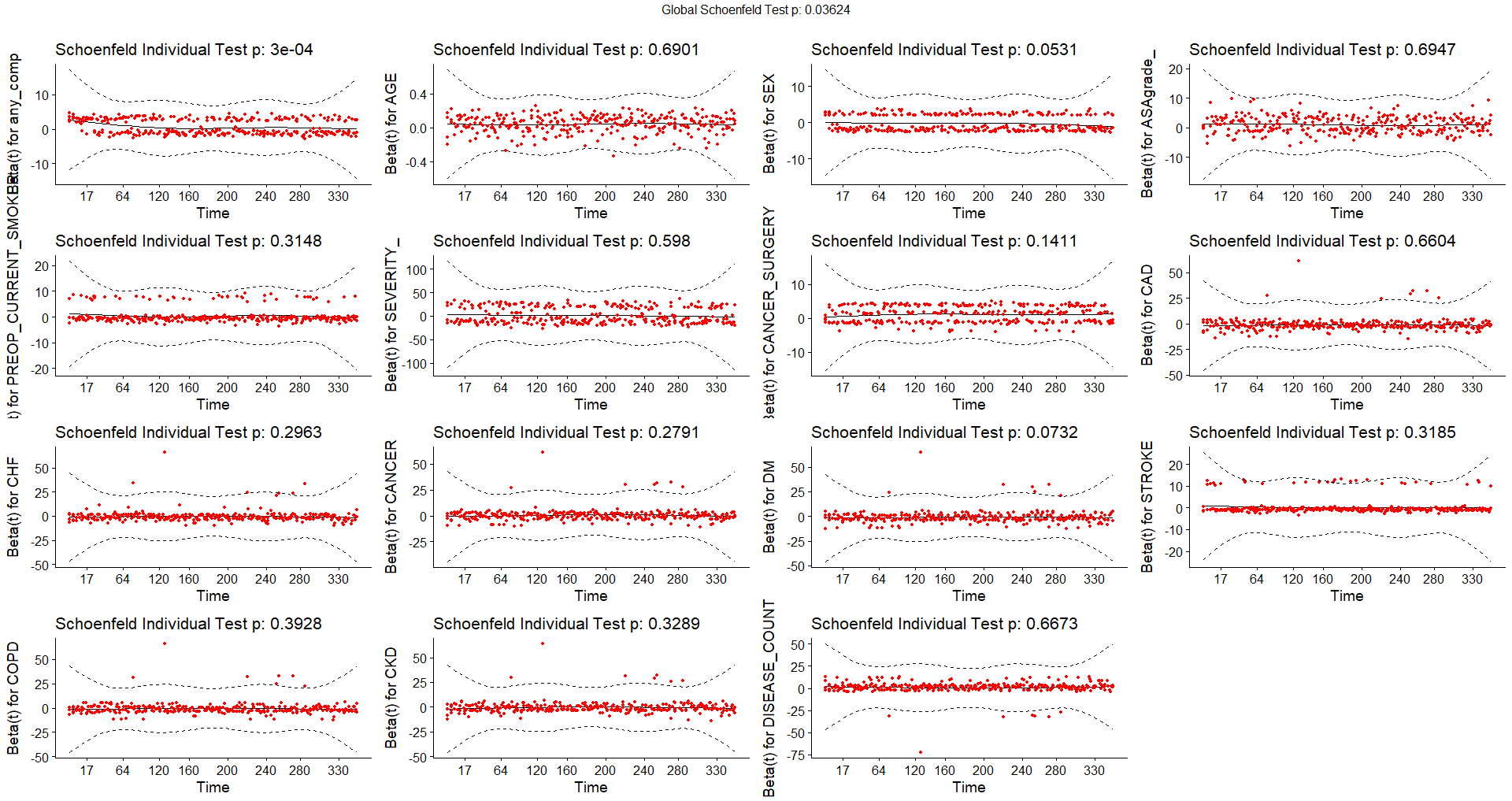


**Supplementary figure 1. Schoenfeld residuals indicating non-proportional hazards in the primary exposure (presence of any complication within 30 days of surgery).**

**
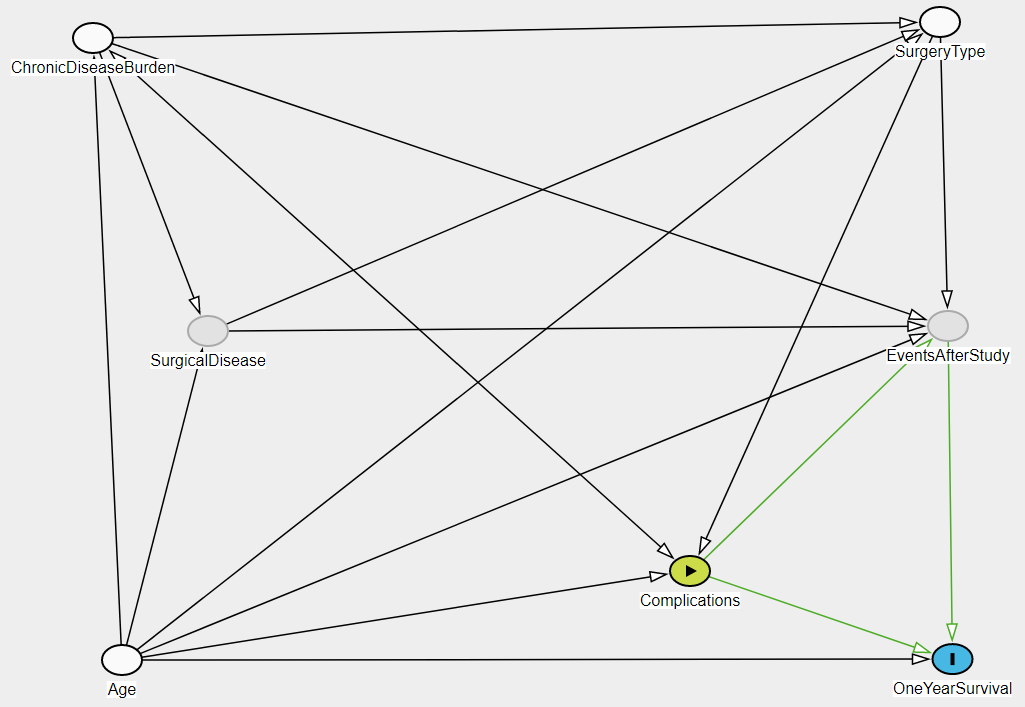
**

**Supplementary figure 2. Directed acyclic graph demonstrating the presumed causal relationships between preoperative variables, complications and one year survival.** Please see code supplement A for a summary of how to re-create and interrogate this DAG.

**
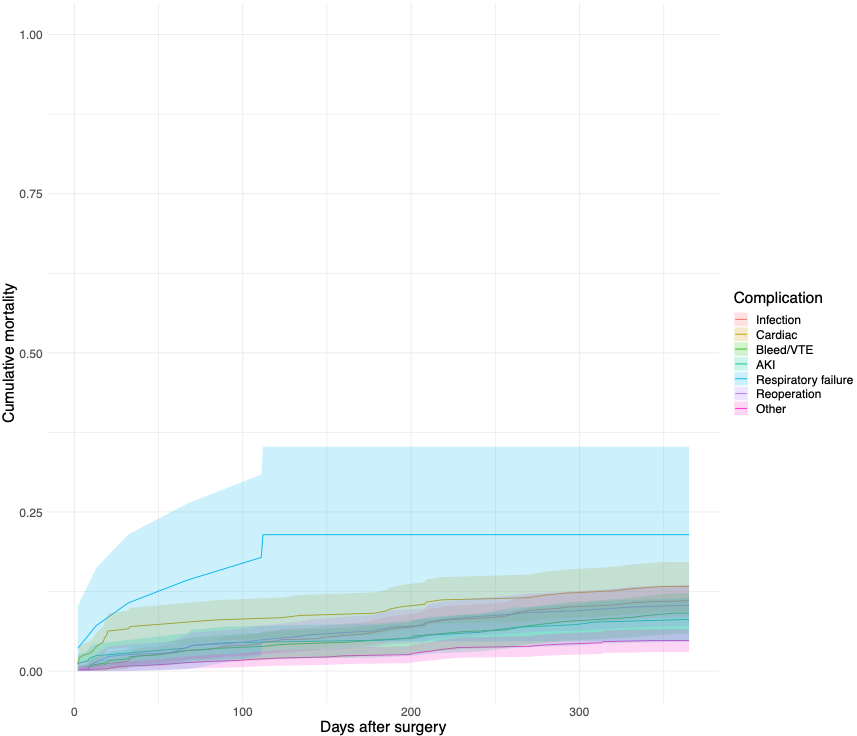
**

**Supplementary figure 3. Cumulative mortality following surgery, stratified by type of complication.** Shaded areas indicate the 95% confidence interval.

**
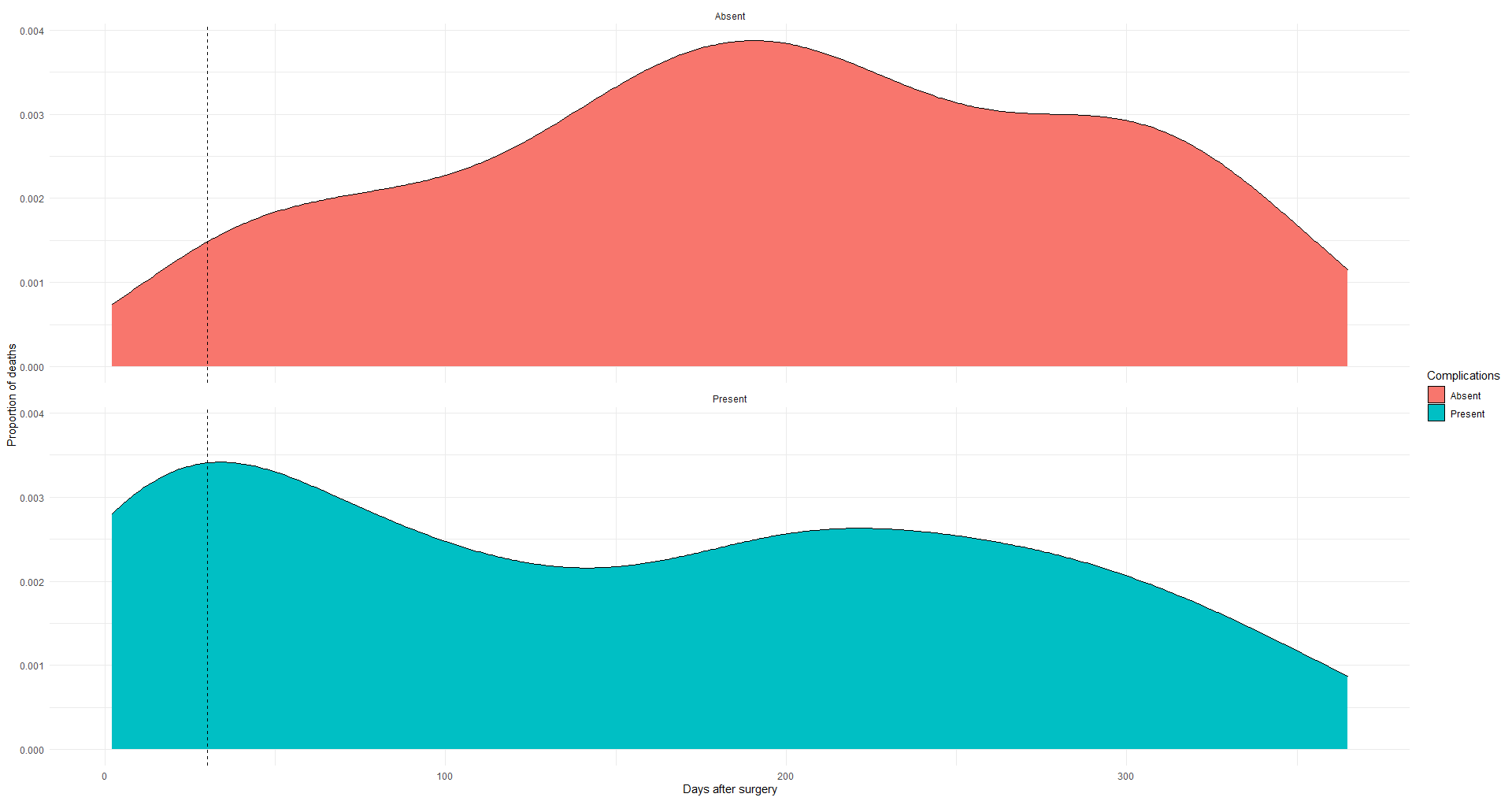
**

**Supplementary figure 4. Density plot of time to death after surgery, stratified by the presence of complications.**

**Code supplement A: Code defining directed acyclic graph in supplementary figure 3. The DAG can be reconstructed and interrogated by visiting:** <https://www.daggity.net> and entering the code into the Model Code interface. Testable assumptions can then be derived from the graph.

dag {

Age [adjusted,pos="-0.070,-0.857"]

ChronicDiseaseBurden [adjusted,pos="-0.070,-0.982"]

Complications [exposure,pos="-0.040,-0.872"]

EventsAfterStudy [latent,pos="-0.029,-0.920"]

OneYearSurvival [outcome,pos="-0.029,-0.855"]

SurgeryType [adjusted,pos="-0.030,-0.979"]

SurgicalDisease [latent,pos="-0.061,-0.919"]

Age -> ChronicDiseaseBurden

Age -> Complications

Age -> EventsAfterStudy

Age -> OneYearSurvival

Age -> SurgeryType

Age -> SurgicalDisease

ChronicDiseaseBurden -> EventsAfterStudy

ChronicDiseaseBurden -> SurgeryType

ChronicDiseaseBurden -> SurgicalDisease

ChronicDiseaseBurden <-> Complications

Complications -> EventsAfterStudy

Complications -> OneYearSurvival

EventsAfterStudy -> OneYearSurvival

SurgeryType -> Complications

SurgeryType -> EventsAfterStudy

SurgicalDisease -> EventsAfterStudy

SurgicalDisease -> SurgeryType

}
